# Creative Motivation and Self-Efficacy Moderate the Differences in Individual Creativity Performance in Interactive Situations

**DOI:** 10.3390/bs16040512

**Published:** 2026-03-29

**Authors:** Ching-Lin Wu

**Affiliations:** Program of Learning Sciences, National Taiwan Normal University, Taipei 106308, Taiwan; chilin570@ntnu.edu.tw

**Keywords:** creative motivation, creative self-efficacy, divergent thinking, remote associates test, online creativity task

## Abstract

The present study examined how creative intrinsic motivation (CIM), creative extrinsic motivation (CEM), and creative self-efficacy (CSE) moderate differences in individual creativity in one-on-one interactive situations. A total of 262 adults completed the Alternative Uses Task and Chinese Radical Remote Associates Test in single- and paired-player modes on an online interactive creativity task platform, followed by measures of CIM, CEM, and CSE. Participants were classified as relatively higher- versus lower-performing members within each dyad on the basis of their single-player performance. The results showed that CIM and CSE significantly moderated the fluency and originality advantages of higher divergent-thinking performers in the paired-player mode, whereas CEM did not significantly moderate performance. No significant moderating effects were found for CRRAT performance. These findings suggest that individual differences in creative motivation and creative self-efficacy are especially relevant when open-ended creative performance unfolds in interactive settings. They also imply that educators and facilitators seeking to improve collaborative creativity should attend to baseline creative ability, as well as learners’ intrinsic motivation and confidence in their creative capabilities.

## 1. Introduction

Creativity is a fundamental psychological capacity that supports adaptive thinking and problem-solving across educational and social contexts (Yalçın & Erden, 2021; Yeh et al., 2019). Rather than being a unitary construct, creativity has been conceptualized differently across theoretical traditions, thereby reflecting variations in research focus and methodological orientation (Runco, 2007). From a psychological perspective, creativity is a dynamic cognitive process through which individuals construct ideas or solutions that simultaneously satisfy the criteria of originality and appropriateness. This process is supported by the integrated engagement of divergent and convergent thinking mechanisms (Glăveanu, 2018).

In empirical research, creative problem-solving is frequently operationalized using tasks that differ in structural openness (Lin et al., 2012; Lin & Lien, 2013). Open-ended tasks, such as the Alternative Uses Task (AUT; Guilford, 1956; Kenett & Faust, 2019), are designed to assess variability in ideational exploration, emphasizing individual differences in originality and flexibility. In contrast, closed-ended tasks, including the Remote Associates Test (RAT; Mednick, 1968; Marko et al., 2019; C. L. Wu & Chen, 2017), assess the ability to integrate remote semantic associations to reach a single target solution. Recent developments in psychological assessment have facilitated the transition of these tasks to digital environments that increasingly employ automated scoring algorithms (Beaty & Johnson, 2021; Zedelius et al., 2019). This methodological shift has not only enhanced efficiency and scalability but has also enabled more fine-grained analyses of cognitive processes underlying creative performance.

Beyond studies that examine creativity as an individual trait or emergent property of groups (Coursey et al., 2019, 2020; Łucznik & May, 2021; Oztop & Gummerum, 2020; C. L. Wu et al., 2020b; C. L. Wu & Chen, 2021), contemporary psychological research has increasingly emphasized the situational nature of creative expression, particularly in collaborative contexts (Sun et al., 2021; C. L. Wu & Chen, 2022). Therefore, individual creativity is not merely a function of cognitive ability but is shaped by social interaction and internal psychological states during group engagement. Because creative work in educational and professional settings is increasingly accomplished through collaboration rather than in isolation, understanding how individuals contribute within interactive contexts has become important for educational psychology and broader innovation-related practices (Acar et al., 2023; Coursey et al., 2019). A key question, therefore, concerns the psychological factors that account for variability in individuals’ creative contributions during collaborative tasks.

The motivational and self-belief constructs are particularly influential. Creative motivation (CM; X. Zhang et al., 2021) reflects an individual’s inclination to initiate and sustain engagement in the creative activity, whereas creative self-efficacy (CSE; Haase et al., 2018) refers to beliefs about one’s capability to produce creative outcomes. Drawing on social cognitive theory and related work on efficacy beliefs and motivated action, these constructs are expected to influence creative performance by shaping effort allocation, persistence in the face of uncertainty, and responsiveness to social cues in group settings (Bandura, 1997; Liu et al., 2025). Examining the roles of CM and CSE in collaborative creative problem-solving can advance the understanding of the psychological mechanisms that facilitate individual creativity in social contexts, with implications for both theory development and creativity-oriented educational interventions.

### 1.1. Creativity Performance in Interactive Situations

Creativity assessment varies substantially, depending on the theoretical assumptions and the context of examination. In psychological research on individual creativity, divergent thinking tasks, most notably the Alternative Uses Task (AUT), are frequently employed to capture ideational exploration and generative capacity (Torrance, 1974). In contrast, insight-based problem-solving and the Remote Associates Test (RAT) are commonly used to assess performance on closed-ended creative tasks that require integrative reasoning and representational restructuring (Lin & Lien, 2013). When creativity is examined in a group context, researchers tend to rely on open-ended tasks that allow multiple individuals to jointly construct ideas through interaction and coordination. Such tasks have been implemented across a range of domains, including drama writing (Zeilig et al., 2018), digital storytelling (Schmoelz, 2018), creative dance (Łucznik & May, 2021), and mathematical problem-solving (Molad et al., 2020), all of which emphasize the dynamic exchange of ideas among group members.

Existing research on collaborative and collective creativity has shown that interactions among dyads, small groups, and teams can facilitate idea generation, elaboration, and innovation. Such benefits, however, depend on group composition, interaction structure, and the nature of the task (Acar et al., 2023). For example, studies of collaborative ideation have shown that expertise diversity and elaborative exchanges can support the linkage between divergent and convergent group creativity (Coursey et al., 2019), whereas work in mathematical and artistic contexts has highlighted how small-group interaction may foster collective originality, flexibility, and shared flow experiences (Levenson & Molad, 2022; Łucznik & May, 2021). Simultaneously, this literature has focused primarily on group-level products, collective processes, or shared outcomes rather than on how individual members perform differently within interactive settings. This study extends this line of research by examining individual creativity performance under paired-player conditions using standardized tasks that permit direct comparisons with single-player performances. In this way, the study addresses a more specific question: How do stable individual characteristics, such as creative motivation and creative self-efficacy, shape individual creative performance when creativity unfolds in interaction rather than in isolation?

From a psychological measurement perspective, individual creative contributions during collaborative activities can be inferred by comparing their performance in joint and independent tasks. However, direct comparison of creativity expressed in individual and collaborative settings poses methodological challenges as group processes may obscure individual-level differences. One approach to address this issue is to compare individuals’ performance on standardized creativity measures, such as divergent thinking tasks and RATs, under one-on-one interactive conditions with their performance in individual testing situations. This design enables the examination of the impact of interpersonal interaction in modulating individual creative expressions while maintaining comparability across conditions. Therefore, the present study utilized widely used individual creativity assessment tools.

### 1.2. Divergent and Convergent Measures of Creativity

Divergent thinking and remote associative tasks capture partially distinct cognitive demands and may respond differently to interactive conditions (Lin & Lien, 2013). Prior work has emphasized that different creativity measures often reflect varied aspects of creative potential and performance, highlighting the importance of theoretical alignment between constructs and assessments (Reiter-Palmon & Schoenbeck, 2020). The construct of divergent thinking originates from Guilford’s (1956) structure-of-intellect framework and is typically operationalized through multiple dimensions that reflect different aspects of ideational production. These dimensions include fluency or the quantity of ideas generated; flexibility, referring to the diversity of conceptual categories represented; originality, which captures the novelty and appropriateness of responses; elaboration, which is defined as the extent to which ideas are developed with additional detail (Kenett & Faust, 2019). Collectively, divergent thinking reflects an individual’s capacity to generate a wide range of novel ideas across multiple conceptual pathways in response to an open-ended stimulus.

Among the divergent thinking measures, the Torrance Tests of Creative Thinking (TTCT) remain one of the most extensively applied instruments in creativity research (Torrance, 1974). The TTCT includes verbal and figural forms, each of which comprises several subtests designed to elicit creative responses through different representational modalities. In the Taiwanese context, the Chinese Version of the Creative Thinking Test (CVCTT; C. C. Wu et al., 1998) has been widely adopted in empirical studies of divergent thinking (Chang et al., 2017; Chen et al., 2020; C. L. Wu et al., 2017). Similar to the TTCT, the CVCTT contains verbal and figural components. The verbal component functions as an AUT, requiring respondents to generate unconventional uses for bamboo chopsticks, whereas the figural component asks participants to produce drawings based on the visual form of the Chinese character “人” (jen; human). The CVCTT-Verbal assesses fluency, flexibility, and originality, whereas the CVCTT-Figural captures elaboration, allowing for a multidimensional evaluation of divergent thinking performance.

In contrast to open-ended ideational tasks, closed-ended creative problem-solving involves situations in which individuals must converge on a single correct solution to an ill-defined or abstract problem (Lin et al., 2012). Successful performance in such tasks typically requires restructuring the initial problem representation to overcome cognitive impasses, a process often accompanied by sudden insight or an “aha” experience (Weisberg, 2015). Divergent thinking emphasizes the generation of multiple possibilities, while closed-ended creative problem-solving involves selectively integrating limited cues to identify an optimal solution.

The RAT represents a prototypical measure of closed-ended creative problem-solving. Each RAT item comprises three seemingly unrelated stimulus words, and respondents are required to identify a fourth word that forms a meaningful association with all three (Mednick, 1968). For example, given the stimuli “bass,” “complex,” and “sleep,” the solution “deep” can be combined with each word to form familiar expressions. To accommodate the linguistic characteristics specific to Chinese, Jen et al. (2004) developed the Chinese Remote Associates Test (CRAT), in which each item is composed of three Chinese characters. For instance, the characters 童 (child), 輕 (light), and 去 (go) can be meaningfully combined with 年 (year) to form compound words. Subsequent works by Jen et al. (2004), Huang et al. (2012), Chang et al. (2016), and C. L. Wu et al. (2017) further expanded this framework by developing RAT variants based on Chinese radicals, characters, and words, resulting in the Chinese Radical RAT (CRRAT), Chinese Compound RAT (CCRAT), and Chinese Word RAT (CWRAT). Due to their relatively short administration times and objective scoring criteria, RAT-based measures have been widely employed in creativity research (C. L. Wu et al., 2020a).

Thus, the AUT and RAT are widely regarded as representative measures of open- and closed-ended creative problem-solving, respectively (C. L. Wu & Chen, 2022; C.-L. Wu et al., 2022). When these tasks are completed in a one-on-one interactive setting, individuals may experience problem-solving processes that differ from those observed in individual conditions. In divergent thinking tasks, exposure to a partner’s ideas may facilitate associative activation and stimulate the generation of original responses across multiple categories, although this may also lead to imitation or social comparison effects (Sun et al., 2021). In some cases, individuals may reduce their response output if they perceive that their partner has already provided sufficient ideas, resulting in decreased motivation to contribute additional responses (C. L. Wu, 2022). By contrast, during RAT performance, access to a partner’s solutions may help individuals overcome impasses more efficiently, allowing them to allocate cognitive resources to other items, thereby improving overall performance. However, the benefits of interaction are contingent on the quality of the partner’s contributions, and limited or incorrect suggestions may provide little facilitative value (C. L. Wu & Chen, 2022). Moreover, creative performance in interactive contexts is likely to be influenced by stable individual characteristics, including creative motivation and self-efficacy.

### 1.3. Creative Motivation in Interactive Creativity

Motivation is commonly conceptualized as a multifaceted construct encompassing both intrinsic and extrinsic regulatory processes (Ryan & Deci, 2000). In creativity research, motivational factors, particularly those related to intrinsic regulation, have been repeatedly identified as central determinants of creative engagement and performance (Hennessey, 2010; Hong et al., 2014, 2016; X. Zhang et al., 2021). Recent evidence from online learning contexts further suggests that intrinsic motivation is positively associated with creative idea generation, partly through its relationship with student engagement in the task (Wang, 2022). Intrinsic motivation reflects engagement in an activity driven by inherent interest or enjoyment, whereas extrinsic motivation involves goal-directed behavior oriented toward outcomes that are separate from the activity itself, such as rewards and external evaluations (Deci & Ryan, 2002). Importantly, intrinsic and extrinsic motivational processes are not mutually exclusive, but are dynamically interrelated (Amabile et al., 1994). For instance, individuals may initially engage in a task out of intrinsic interest but subsequently experience shifts in motivational orientation when external incentives or evaluative feedback are introduced, potentially altering the balance between intrinsic and extrinsic regulation (Deci et al., 1996).

A substantial body of psychological research has demonstrated that intrinsic motivation facilitates individual creative performance, particularly in tasks requiring open-ended ideation. Empirical evidence indicates that higher intrinsic motivation is associated with superior performance in divergent thinking tasks, including increased fluency, flexibility, and originality (Hong et al., 2014). These findings suggest that intrinsic interest enhances sustained cognitive exploration and supports the generation of novel ideas across multiple categories. Similarly, research on creative writing has demonstrated that individuals with stronger intrinsic motivation produce more original and expressive output, likely because of their greater cognitive immersion in the task (Hong et al., 2016). In contrast, intrinsic motivation plays a more limited role in closed-ended creative problem-solving, with some studies reporting no significant association between intrinsic motivation and performance on insight-based tasks such as the RAT (Wieth & Burns, 2000). Intrinsic motivation may be stronger when creative activities are embedded in collaborative or online contexts. For example, X. Zhang et al. (2021) found that groups characterized by higher intrinsic motivation demonstrated greater ideational fluency and flexibility during online collaborative tasks and devoted more time to evaluating alternative ideas. Similarly, Wang (2022) reported that students’ intrinsic motivation was positively associated with online creative idea generation, as reflected in greater task engagement.

However, findings on extrinsic motivation and creativity are inconsistent. Some studies have suggested that extrinsic motivation may constrain creative performance by narrowing attentional focus or increasing evaluative pressure (Prabhu et al., 2008), whereas others have indicated that certain forms of extrinsic motivation can enhance creativity under specific conditions (Ochse, 1990). From a psychological perspective, the effect of extrinsic motivation depends on its functional characteristics. As proposed by Amabile (1996), controlling forms of extrinsic motivation, such as competitive pressure, negative evaluation, and excessive emphasis on rewards, tend to undermine creativity by reducing autonomy. In contrast, informational or enabling extrinsic motivators, including positive feedback and recognition of competence, may support creative performance by reinforcing perceived ability and task engagement (Hung et al., 2008). Concurrently, expected rewards have been shown to facilitate performance in closed-ended creative tasks such as the RAT, suggesting that extrinsic incentives may enhance goal-directed associative processing in insight problem-solving (Cristofori et al., 2018). Collectively, these findings imply that intrinsic and extrinsic motivational processes may be differentially related to open- and closed-ended forms of creative cognition.

### 1.4. Creative Self-Efficacy in Interactive Creativity

Beyond motivational factors, creative self-efficacy (CSE) represents a critical self-referential construct in creativity research. CSE reflects individuals’ beliefs about their capability to generate creative outcomes and can be conceptualized as a domain-specific application of the self-efficacy theory (Bandura, 1997; Hung, 2018). According to social-cognitive theory, self-efficacy influences how individuals approach tasks, regulate efforts, and persist during difficulties (Lemons, 2010). In creative contexts, individuals with stronger CSE are more likely to engage confidently in creative tasks, explore unconventional ideas, and sustain efforts when encountering uncertainty, thereby increasing the likelihood of successful creative performance (Tierney & Farmer, 2002). Recent studies continue to support the relevance of CSE in educational contexts, showing that stronger creative self-efficacy is associated with more adaptive responses to feedback and higher-order problem-solving processes (Abulela, 2024; Liu et al., 2025).

Empirical research has consistently demonstrated the positive association between CSE and creative outcomes. Higher CSE predict better performance on divergent thinking tasks, particularly in terms of fluency and originality, indicating that confidence in one’s creative ability facilitates ideational productivity (Ghorbani et al., 2012; Fouladi & Shahidi, 2016; Puente-Díaz & Cavazos-Arroyo, 2017). Meta-analytic evidence supports this relationship, although the strength of the association varies depending on the measurement approach used to assess creativity (Haase et al., 2018). Specifically, CSE showed a stronger correlation with self-reported creativity than with performance-based measures, including divergent thinking, verbal creativity, and figural creativity tasks. These findings underscore the importance of considering measurement characteristics when interpreting the relationship between self-belief and creative performance. Recently, Liu et al. (2025) showed that the effects of creative self-efficacy on creative performance may vary depending on task conditions, particularly when individuals respond to evaluative feedback, suggesting that CSE is best understood as a context-sensitive facilitator rather than a uniformly strong predictor across all creative tasks.

In contrast, the role of CSE in closed-ended creative problem-solving appears to be more limited. Several empirical studies have reported insignificant associations between CSE and RAT performance (Sun et al., 2021; H. Zhang et al., 2020), suggesting that confidence in one’s creative ability may not directly translate into improved performance on tasks that require convergent insight rather than ideational exploration. This pattern indicates that CSE may differentially influence the distinct cognitive processes underlying open- and closed-ended creativity.

In summary, both creative motivation and creative self-efficacy are important psychological correlates of creativity; however, their effects vary across task types. Intrinsic motivation and CSE reliably predict performance in divergent thinking tasks (Fouladi & Shahidi, 2016; X. Zhang et al., 2021), whereas extrinsic motivation demonstrates more context-dependent effects (Prabhu et al., 2008). In contrast, closed-ended creative problem-solving appears less sensitive to intrinsic motivation and CSE even though certain forms of extrinsic motivation may facilitate performance (Cristofori et al., 2018; Wieth & Burns, 2000). Thus, the present study further examined whether interaction in one-on-one collaborative settings can attenuate performance differences between individuals with lower baseline creativity but higher motivational or self-efficacy resources and those with higher baseline creativity, thereby elucidating the conditions under which psychological characteristics shape creative outcomes in social contexts.

### 1.5. The Present Study

Empirical findings suggest that individual differences in creative motivation and self-related beliefs are systematically associated with creative cognitive performance. Specifically, creative intrinsic motivation (CIM) and creative self-efficacy (CSE) are positively related to divergent thinking indices, including fluency, flexibility, and originality (Haase et al., 2018; Hong et al., 2014). Additionally, creative extrinsic motivation (CEM) is associated with performance in remote associative tasks, indicating its relevance for closed-ended creative problem-solving (Cristofori et al., 2018). However, creative performance is subject to practical constraints such as limited response time and a fixed number of test items, which may restrict the expression of individual differences under standard testing conditions. These constraints highlight the potential importance of interactive contexts, in which social input may amplify or attenuate the expression of underlying creative capacities.

From a social-cognitive perspective, motivational and self-efficacy factors are expected to shape how individuals capitalize on interactive opportunities during creative tasks. Accordingly, the present study proposes that CIM and CSE function as moderating variables in paired-player divergent thinking tasks, influencing the extent to which interactions enhance creative output. Specifically, individuals with relatively strong divergent thinking abilities are expected to demonstrate higher fluency, flexibility, and originality in interactive settings when they have high levels of CIM or CSE. In contrast, when CIM or CSE is low, the performance advantage associated with higher divergent thinking ability is expected to be reduced, as individuals may be less inclined to fully engage with their partner’s ideas or persist in ideational exploration.

A similar moderating mechanism is proposed for closed-ended creative problem-solving. Considering the previously identified association between CEM and remote associative performance (Cristofori et al., 2018), the present study hypothesized that CEM moderates performance on the Chinese Radical Remote Associates Test (CRRAT) under paired-player conditions. Specifically, individuals with stronger remote associative abilities were expected to achieve superior CRRAT performance during interactions when they report higher levels of CEM. Conversely, when CEM is low, differences in CRRAT performance between individuals with high and low remote associative ability are expected to diminish because extrinsic motivational incentives may be insufficient to facilitate the effective use of partner-provided cues in the problem-solving process.

The present study addressed the following research questions to explicitly state the study’s purpose:

**RQ1.**  *Do higher- and lower-performing individuals differ in their creative performance under paired-player conditions*?

**RQ2.**  *Are CIM, CEM, and CSE associated with paired-player performance on divergent thinking and CRRAT tasks*?

**RQ3.**  *Do CIM, CEM, and CSE moderate the relationship between baseline individual creative performance and creative outcomes in one-on-one interactive situations*?

Based on the prior literature, the following hypotheses were proposed:

**H1.**  *Individuals with relatively higher divergent-thinking ability in the single-player mode outperform their lower-performing partners in terms of fluency, flexibility, and originality in the paired-player mode*.

**H2.**  *CIM and CSE positively moderate the relationship between baseline divergent thinking performance and paired-player divergent thinking outcomes, such that the advantage of higher-performing individuals is stronger when CIM or CSE is high*.

**H3.**  *CEM positively moderates the relationship between baseline CRRAT performance and paired-player CRRAT outcomes*.

## 2. Methods

### 2.1. Participants

An a priori power analysis indicated that a minimum of 119 participants was required to detect a medium effect size (f^2^ = 0.15) at α =.05 with statistical power of.95. The final sample comprised 262 adults (185 women and 77 men) aged 20–30 years (M = 23.27, SD = 2.67). Participants were recruited through university-based announcements and online recruitment messages targeting young adult native Chinese speakers who could complete computer-based creativity tasks in a laboratory setting. Most participants were college students or recent graduates, which should be considered when interpreting the generalizability of the findings. The study was approved by the Institutional Review Board of the National Taiwan Normal University, and all participants provided written informed consent prior to participation. Each participant received NT$300 upon task completion.

### 2.2. Online Creativity Task

The online creativity task comprised two computerized activities designed to assess open-ended and closed-ended creative problem solving: a divergent thinking test based on the Alternative Uses Task (AUT) and the Chinese Radical Remote Associates Test (CRRAT; C.-L. Wu et al., 2022). The task interface was structured to support both individual and interactive testing conditions, and included distinct areas for stimulus presentation, response display, response input, remaining time, and mode indication (see Appendix A for a sample screenshot of the interface). Participants completed the tasks independently or in paired-player mode. In the paired-player condition, two participants with consecutive identification numbers (e.g., Participants 2 and 3) were assigned as a dyad to complete the creativity tasks simultaneously. When one participant submitted a response, the partner could view the response in real time and decide whether to incorporate it into their problem-solving process. Consistency was ensured across task types by pairing the participants with the same partner for both creative tasks under the paired-player condition.

#### 2.2.1. Divergent Thinking Test

The divergent thinking test required participants to generate unconventional uses for two everyday objects: plastic bottles and drinking straws. Performance was evaluated based on the three standard dimensions of divergent thinking, including fluency, flexibility, and originality, which were calculated by the system automatically. The reliability of the automated scoring procedure was examined by comparing computer-generated scores with manual ratings, which yielded a high correspondence across the indices (*r*s = 0.99, 0.92, 0.97, 0.97, 0.92, and 0.95). Evidence of criterion-related validity was demonstrated through moderate to strong correlations with established AUT measures involving bamboo chopsticks (C. C. Wu et al., 1998) and newspapers (Hsu et al., 2012; *r*s = 0.79, 0.54, 0.58, 0.75, 0.51, and 0.60). Additionally, low correlations with the CRRAT scores (*r*s = 0.05 to 0.18) provided support for discriminant validity, indicating that the divergent thinking task captured cognitive processes distinct from those involved in closed-ended creative problem-solving.

#### 2.2.2. CRRAT

The Chinese Radical Remote Associates Test (CRRAT) was used to assess closed-ended creative problem-solving. The CRRAT comprises two parallel forms developed by Chang et al. (2016), each containing 20 items. For each item, the participants are presented with three Chinese radicals and they are required to identify an additional radical that could be combined with each stimulus radical to form three meaningful Chinese characters. For example, the radicals 女 (female), 子 (son), and 禾 (rice seedling) can all be paired with the radical 乃 (be) to generate valid characters. Each accurately solved item is awarded one point. The sum of the correct responses forms the total score.

Psychometric evaluation indicated that the CRRAT demonstrated satisfactory internal consistency across both forms (Cronbach’s α = 0.80 and 0.79). Criterion-related validity was supported by moderate correlations with measures of insight problem-solving (*r*s = 0.48 and 0.38) and the Chinese Word Remote Associates Test (CWRAT; *r*s = 0.58 and 0.48; C.-L. Wu et al., 2022). Therefore, the CRRAT reliably depicts individual differences in remote associative abilities and convergent creative reasoning.

### 2.3. Measures

#### 2.3.1. Creative Intrinsic Motivation Scale

The creative intrinsic motivation scale contains five subscales: ability, mood for playfulness, preference for complexity, autonomous decision-making, and work engagement. It includes 17 items rated on a four-point scale. Higher scores indicate higher levels of intrinsic motivation when undertaking a creativity task (Hung et al., 2008).

The internal consistency reliability for the scale was 0.91 and it ranged between 0.75 and 0.81 for the subscales. Regarding validity, CIM had significant correlations with all dimensions of the creative life experience scale (*r* = 0.27–0.62, *p* < 0.001), including scientific and creative problem-solving, applying new knowledge and tirelessly seeking improvement, performing arts innovation, design of visual life, change in lifestyle, open-mindedness, making surprises, old bottles with new wine, and computer programming. The outcome of the five-factor orthogonal model was acceptable (RMR = 0.02, RMSEA = 0.06, GFI = 0.94, and AGFI = 0.92), indicating a good factor structure.

#### 2.3.2. Creative Extrinsic Motivation Scale

The creative extrinsic motivation scale comprises 17 items rated on a four-point scale, including the dimensions of winning recognition, valuing competition, external rewards, and others’ arrangements. Higher scores indicate higher levels of extrinsic motivation when engaging in creative thinking (Hung et al., 2008).

The internal consistency reliability of the overall scale was 0.83, whereas it ranged between 0.67 and 0.90 for its subscales. Regarding validity, extrinsic motivation had no significant correlations with the following dimensions of the creative life experience scale: change in lifestyle, open-mindedness, and computer programming; however, it had significant correlations with the remaining dimensions (*r* = 0.19–0.24, *p* < 0.001). The four-factor orthogonal model was acceptable (RMR = 0.02, RMSEA = 0.06, GFI = 0.93, and AGFI = 0.90), indicating a good factor structure. The factor loadings of the items ranged between 0.41 and 0.89. The coefficients of determination of each item for the overall model ranged between 0.29 and 0.72, indicating that each test item had good quality.

#### 2.3.3. Creative Self-Efficacy Scale

The creative self-efficacy scale comprises 12 questions covering three dimensions: belief in creative thinking strategies, production of creative products, and ability to resist negative evaluations. Each item is rated on a four-point Likert scale. Higher scores indicate higher CSE. This information has been compiled by Hung et al. (2008).

The internal consistency reliability of the overall scale was 0.85, while it ranged between 0.72 and 0.83 for each subscale, indicating good internal consistency. Its test–retest reliability was 0.88 with a two-week interval. Correlations between the subscales and the CSE scale ranged from 0.71 to 0.85. Concerning validity, the outcome of the three-factor orthogonal model was acceptable (RMR = 0.02, RMSEA = 0.05, GFI = 0.96, and AGFI = 0.95), indicating a good factor structure. The factor loadings of its items were between 0.54 and 0.85, and the coefficients of determination for each item to the whole model were 0.29 and 0.75, indicating that each item had good quality.

### 2.4. Procedure

The experimental sessions were conducted in a computer-based classroom setting with up to five dyads completing the tasks concurrently in each session. Participants were randomly assigned to paired groups for the interactive condition. The creativity tasks were completed under controlled laboratory conditions. Before task administration, the researcher provided a standardized explanation of the study objectives and procedural flow, after which all participants provided their written informed consent in accordance with the ethical research guidelines.

Thereafter, the participants completed creativity tasks via an online interactive creativity platform. The task battery included two divergent thinking tasks, including unusual uses of plastic bottles and drinking straws, and two forms of the Chinese Radical Remote Associates Test (CRRAT A and CRRAT B). Each task was performed within a fixed time duration of 10 min. The presentation order of the four tasks was counterbalanced across participants to minimize the order and learning effects. Moreover, the participants completed different tasks under single- and paired-player conditions, allowing for within-subject comparisons of individual and interactive performances. For example, some participants completed the unusual uses of the plastic bottles task and CRRAT A individually, whereas they completed the unusual uses of the straw task and CRRAT B in the paired-player condition; this assignment was systematically rotated across participants.

Upon task completion, the participants responded to a series of self-report questionnaires assessing creative intrinsic motivation (CIM), creative extrinsic motivation (CEM), and creative self-efficacy (CSE). The completion of the motivational and self-efficacy measures required approximately 15 min. This sequence ensured that responses to the self-report instruments were not influenced by anticipatory effects prior to task engagement.

### 2.5. Data Analysis

Participants’ creative performance was quantified across divergent and convergent task domains. For the divergent thinking tasks, fluency, flexibility, and originality scores were computed separately for each task version. Performance on the CRRAT was indexed by the total number of correct responses for Forms A and B. Raw scores for fluency, flexibility, and originality were first standardized into z-scores and subsequently transformed into T-scores using the formula, T = 50 + 10 × z, to enable aggregation across divergent thinking dimensions while controlling for scale differences. The resulting T-scores were then summed to form a composite divergent thinking index consistent with the established scoring procedures (C. C. Wu et al., 1998).

To examine relative performance differences within interactional units, participants were classified within each dyad rather than by an absolute sample-wide cutoff. Specifically, for each task domain, the member with the higher single-player score was labeled as the relatively higher-performing member, and the partner was labeled as the relatively lower-performing member. This grouping strategy was adopted because this study focused on within-dyad asymmetry in baseline ability, a theoretically relevant construct for one-on-one interactive performance. Accordingly, the analytic grouping variable represented the relative individual standing within a dyad, not a characteristic of the dyad as a whole. Therefore, this categorization should be interpreted as an analytic device for examining relative asymmetry within dyads rather than as a fixed classification of creative ability. Additionally, mean scores were calculated for creative intrinsic motivation (CIM), creative extrinsic motivation (CEM), and creative self-efficacy (CSE) to represent stable individual differences in motivational and self-belief constructs.

Preliminary analyses included independent-samples *t*-tests to compare creativity performance in the paired-player condition, as well as CIM and CEM levels between the divergent thinking and CRRAT performance groups. Pearson product–moment correlations were computed to examine the associations between creativity performance in the interactive condition and motivational variables (CIM and CEM).

The proposed moderating effects of CIM, CEM, and CSE on the relationship between baseline creativity group membership (divergent thinking or CRRAT performance) and creativity outcomes in the paired-player mode were examined through moderation analyses performed using the PROCESS macro for SPSS 20.0 (Hayes, 2013). A nonparametric bootstrapping procedure was employed to reduce reliance on distributional assumptions and enhance the robustness of the parameter estimates. Specifically, 5000 bootstrap samples were generated by resampling with replacement from the original dataset (N = 262), and regression coefficients and conditional effect estimates were calculated for each resampled dataset. The confidence intervals for the moderating effects were derived from the average of these estimates.

Significant interactions were further analyzed using simple slope analysis. High and low levels of the moderator variables (CIM, CEM, and CSE) were defined as one standard deviation above and below the sample mean, respectively. These analyses examined whether the strength and direction of the association between group membership and paired-player creativity performance differed between conditions of relatively high and low motivation or creative self-efficacy.

## 3. Results

### 3.1. Descriptive Statistics of Creativity Performance in Paired-Player Mode and Creative Motivation

Table 1 displays the mean and standard deviation (SD) of the CIM, CEM, and CSE for the CRAT and divergent thinking groups. The high-*performing* CRAT group (*M* = 0.50, *SD* = 0.18) performed better than the low-*performing* one (*M* = 0.40, *SD* = 0.20) in the paired-player mode (*t* [260] = 4.24, *p* < 0.01, *d* = 0.53). In contrast, there were no significant differences in CIM (*t* [260] = 1.32, *p* = 0.19, *d* = 0.16), CEM (*t* [260] = 0.31, *p* = 0.76, *d* = 0.04), or CSE (*t* [260] = 0.76, *p* = 0.45, *d* = 0.09). For the divergent thinking groups, the high-*performing* group performed significantly better than the low-*performing* group in fluency (*t* [260] = 3.80, *p* < 0.01, *d* = 0.47), flexibility (*t* [260] = 3.50, *p* < 0.01, *d* = 0.43), and originality (*t* [260] = 3.47, *p* < 0.01, *d* = 0.43). However, they did not have noticeable differences in CIM (*t* [260] = 1.55, *p* = 0.12, *d* = 0.19), CEM (*t* [260] = −0.54, *p* = 0.59, *d* = 0.07), and CSE (*t* [260] = 0.39, *p* = 0.70, *d* = 0.05).

### 3.2. Relationship Between Creativity Performance in Paired-Player Mode and Creative Motivation

Table 2 displays the respective correlations of the CRRAT and divergent thinking scores in the paired-player mode with the CIM, CEM and CSE. The results revealed that in the paired-player mode, CRRAT performance had no significant correlation with CEM, CIM, and CSE (*r*s = 0.02, 0.03, 0.03, *ps* = 0.74, 0.65, 0.60). However, the fluency, flexibility, and originality had positive significant correlations with CIM (*r*s = 0.23, 0.22, 0.23, *ps* < 0.01) and CSE (*r*s = 0.19, 0.13, 0.18, *ps* < 0.05), but had no significant correlation with CEM (*r*s = 0.07, 0.07, 0.04, *ps* = 0.28, 0.28, 0.51). Additionally, CIM was not correlated with CEM but was significantly correlated with CSE. In sum, divergent thinking had positive correlations with CIM and CSE, whereas CRRAT had no significant correlation with CIM, CEM, and CSE in the paired-player mode.

### 3.3. Moderation Analyses

**Figure 1:**
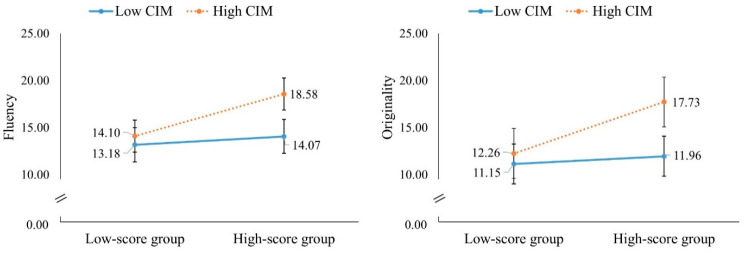
Simple slope effect of CIM on fluency and originality.

Table 3 displays the moderation effects. First, CIM moderated the prediction for the fluency (*β* = 0.28, *t* = 2.38, *p* = 0.02, 95% CI = [0.05, 0.51]) and originality (*β* = 0.28, *t* = 2.38, *p* = 0.02, 95% CI = [0.05, 0.51]) scores of the divergent thinking groups in the paired-player mode. The simple slope analysis revealed that the group with higher divergent thinking had good performance in fluency (*β* = 0.70, *t* = 4.20, *p* < 0.01, 95% CI = [0.37, 1.02]) and originality (*β* = 0.66, *t* = 3.97, *p* < 0.01, 95% CI = [0.33, 0.99]) when they had higher CIM. In contrast, when participants had low CIM, their prior divergent thinking performance did not correctly predict their fluency (*β* = 0.14, *t* = 0.83, *p* = 0.41, 95% CI = [−0.19, 0.47]) and originality (*β* = 0.10, *t* = 0.59, *p* = 0.55, 95% CI = [−0.23, 0.43]) scores in the paired-player mode (Figure 1).

Second, CEM did not moderate the prediction for the fluency (*β* = 0.004, *t* = 0.03, *p* = 0.97, 95% CI = [−0.24, 0.25]), flexibility (*β* = 0.08, *t* = 0.67, *p* = 0.51, 95% CI = [−0.16, 0.32]) and originality (*β* = 0.06, *t* = 0.45, *p* = 0.65, 95% CI = [−0.19, 0.30]) scores of divergent thinking groups in the paired-player mode.

**Figure 2:**
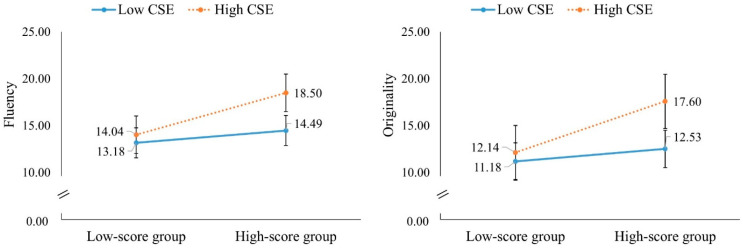
Simple slope effect of CSE on fluency and originality.

Third, CSE moderated the prediction for the fluency (*β* = 0.25, *t* = 2.07, *p* = 0.04, 95% CI = [0.01, 0.48]) and originality (*β* = 0.25, *t* = 2.098, *p* = 0.04, 95% CI = [0.01, 0.48]) scores of the divergent thinking groups in the paired-player mode. The simple slope analysis revealed that the group with higher divergent thinking had good performance in fluency (*β* = 0.69, *t* = 4.16, *p* < 0.01, 95% CI = [0.37, 1.02]) and originality (*β* = 0.66, *t* = 3.94, *p* < 0.01, 95% CI = [0.33, 0.99]) when they had higher CSE. In contrast, when their CSE was low, their divergent thinking performance did not correctly predict their fluency (*β* = 0.20, *t* = 1.22, *p* = 0.22, 95% CI = [−0.13, 0.53]) and originality scores (*β* = 0.16, *t* = 0.97, *p* = 0.33, 95% CI = [−0.17, 0.49]) in the paired-player mode (Figure 2).

Finally, CIM (*β* = −0.01, *t* = −0.05, *p* = 0.96, 95% CI = [−0.24, 0.23]), CEM (*β* = −0.03, *t* = −0.21, *p* = 0.83, 95% CI = [−0.26, 0.21]), and CSE (*β* = 0.04, *t* = 0.29, *p* = 0.77, 95% CI = [−0.20, 0.27]) did not moderate the correlation between CRAT groups and their CRRAT performance in the paired-player mode. In summary, CM and CSE may exert varied moderating effects on different kinds of creativity. 

## 4. Discussion

The present study explored the moderating role of CM and CSE in creativity performance in interactive situations. Therefore, we collected data on participants’ creativity performance in both single- and paired-player modes through an online interactive creativity task platform, in addition to measures of CIM, CEM, and CSE. The research outcome showed that fluency, flexibility, and originality of DT had significant positive correlations with CIM and CSE, but had no association with CEM. In the paired-player mode, CRRAT performance was not significantly correlated with CIM, CEM, or CSE. Additionally, CIM and CSE moderated divergent thinkers’ fluency and originality in the paired-player mode. Those with higher divergent thinking skills tended to have advantages in fluency and originality in the paired-player mode when they had high CIM or CSE. In contrast, CEM did not moderate the CRRAT performance of those with different remote associative abilities in the paired-player mode. In summary, these results revealed the different correlations of CIM, CEM, and CSE with divergent thinking and RAT performance in the paired-player mode and the moderating effect of CIM and CSE on divergent thinking performance in a one-on-one interactive situation.

First, the CRRAT and divergent thinking groups showed significant differences in their performances on the CRRAT and divergent thinking (fluency, flexibility, and originality). This indicates that among the randomly formed two-player groups, those with better creativity performance showed a stable advantage in both the single- and paired-player modes, revealing that the two-player cooperative mode did not eliminate the existing differences between the two respondents in creativity performance. Meanwhile, when considering the two-player groups, those with different levels of creativity showed no significant differences in CM or CSE, indicating that the two group members have homogeneous intrinsic traits. Furthermore, the results showed that those with different levels of creativity differed in their performance on the RAT and divergent thinking in the paired-player mode, which may not be mediated by CM and CSE.

Second, the positive associations between paired-player divergent thinking performance and CIM and CSE are broadly consistent with prior research, which shows that intrinsic motivation and creative self-efficacy support open-ended idea generation and creative engagement (Haase et al., 2018; Hong et al., 2014; X. Zhang et al., 2021). Recent work also suggests that CSE shapes how individuals adaptively respond to challenges and feedback in creative tasks, further supporting the interpretation that efficacy beliefs matter most when tasks allow flexible, self-initiated idea generation (Liu et al., 2025). In contrast, the absence of significant associations or moderating effects for CRRAT performance suggests that closed-ended creative problem-solving may be less sensitive to self-reported motivational and efficacy-related factors in interactive contexts. This pattern partly diverges from the research indicating that reward-related extrinsic motivation can facilitate remote associative performance. However, this discrepancy may be explained by the distinction between experimentally induced reward conditions and trait-like, self-reported extrinsic motivation, which are conceptually related but not identical. Together, these findings suggest that motivational and efficacy-related variables may be more strongly implicated in open-ended creative performance than in closed-ended associative problem-solving, particularly in paired interactive settings.

Third, we discuss the moderating effects of the relevant variables. CIM and CSE can moderate the fluency and originality of individuals with divergent thinking in the paired-player mode. CIM reflects individuals’ positive interest in creative thinking (Hong et al., 2014), whereas CSE represent their subjective belief in creativity (Hung, 2018). Furthermore, the results showed that those with higher divergent thinking have greater advantages in fluency and originality when they have high CIM or CSE. In contrast, when participants had low CIM or CSE, there was no noticeable difference in fluency or originality, regardless of their divergent thinking skills. Therefore, those with highly divergent thinking performed better in fluency and originality in the paired-player mode because of their creativity and willingness to present creative ideas/positive self-concept. Only when they simultaneously manifest these two conditions (i.e., prior creative thinking ability and CIM/CSE) do they display higher creativity in one-on-one interactive situations.

Fourth, CEM did not moderate the performance of individuals with different levels of prior creative ability in the paired-player mode. This finding is consistent with the view that externally induced motivation and internally regulated motivation operate through different psychological mechanisms and may play distinct roles in creative cognition (Ryan & Deci, 2000; Cristofori et al., 2018; X. Zhang et al., 2021). At the same time, the present results should not be interpreted as evidence that extrinsic motivation is irrelevant to creative performance. Rather, they suggest that self-reported extrinsic creative motivation may not be sufficient to differentiate paired-player performance in the present interactive context. This point is particularly important because prior studies showing positive effects of extrinsic motivation on remote associative performance have often relied on experimentally induced rewards rather than trait-like self-reports (e.g., Cristofori et al., 2018). Accordingly, the current findings may reflect a distinction between reward-triggered extrinsic motivation and individuals’ self-perceived extrinsic motivational orientation. Future research should examine whether other forms of extrinsic motivation, such as material rewards, performance-contingent incentives, or enabling contextual supports, exert different effects on creative performance in interactive settings.

Finally, in the paired-player mode, the CRRAT performance of individuals with different levels of prior creative ability was not moderated by creative motivation or creative self-efficacy. Rather, prior creative thinking ability remained a strong predictor of CRRAT performance, suggesting that performance on closed-ended creative problem-solving tasks may depend more heavily on task-specific cognitive ability than on motivational or efficacy-related factors. These findings differ from those on divergent thinking and may be attributed to the distinct cognitive demands of open- and closed-ended creativity tasks (Lin & Lien, 2013). The contrast between divergent thinking and the CRRAT findings also has implications for creativity assessment. Rather than treating creativity as a single undifferentiated construct, the present findings support the view that measurement format influences which aspects of creativity become visible in research (Reiter-Palmon & Schoenbeck, 2020). Open-ended tasks may be particularly sensitive to motivational engagement and efficacy beliefs, whereas closed-ended associative tasks may reflect task-specific cognitive efficiency or prior abilities more strongly. In divergent thinking tasks, individuals can generate multiple acceptable responses and may therefore benefit more from stronger motivation and greater confidence in their creative capability. In contrast, the CRRAT items require the retrieval of a specific correct answer, which may limit the extent to which motivation and self-efficacy directly facilitate performance. These findings suggest that motivational- and efficacy-related factors may play a more prominent role in open-ended creative production than in closed-ended associative problem solving, particularly in paired interactive contexts.

The present study had some limitations. First, this study utilized self-reported measures to assess CIM, CEM, and CSE; CEM did not have significant correlations with the two types of creativity. This finding differs from those of previous studies that assessed participants’ extrinsic motivation using material rewards (Cristofori et al., 2018). Therefore, future research should utilize different methods to analyze CIM, CEM, and CSE at both individual and group levels. Additionally, the higher/lower grouping was based on relative within-dyad differences, and some dyads may have been classified despite minimal score gaps. Future research could adopt minimum-difference criteria, latent profile approaches, or continuous interaction models to assess whether the same moderation patterns hold when the baseline performance is modeled without dichotomization. Finally, the online creativity task platform used in this study allowed participants to read the response(s) of other group members when they performed the tasks (indirect interaction); however, there were no direct interactions during the process. Future research could compare the impact of indirect and direct interactive patterns on creativity performance and analyze the group performance.

Furthermore, the present study used online interactive standardized creativity measurements to more comprehensively explore the moderating role of CIM, CEM, and CSE in open- and closed-ended creativity performance in a one-on-one interactive situation and deepen the understanding of individual creativity performance in a group. The present findings have several implications for educational practice and creative research. First, they suggest that individual performance in interactive creativity tasks is shaped by baseline creative ability and motivational and efficacy-related resources, particularly intrinsic motivation and creative self-efficacy. This indicates that collaborative or pair-based creative activities may not be sufficient to enhance creative performance unless learners are supported in sustaining interest in the task and confidence in their creative capabilities. Second, the differential pattern observed between divergent-thinking and CRRAT performance suggests that the effects of motivational factors may vary with task characteristics. Accordingly, both instructional design and creativity assessment should be aligned with the specific cognitive and psychological demands of the task, rather than assuming that different forms of creative performance are equally responsive to the same set of individual factors.

In conclusion, the present study shows that individual creativity in interactive settings is shaped not only by prior ability but also by motivational and efficacy-related resources. Particularly, intrinsic motivation and creative self-efficacy appear to strengthen the performance advantages of relatively stronger divergent thinkers in paired interactions. In contrast, closed-ended associative problem solving appears more strongly constrained by prior task-specific ability. These findings underscore the important educational implication that interactive creativity does not automatically arise from social contact alone. Instead, learners are more likely to contribute creatively when competence-related and motivational resources support the collaboration.

## Figures and Tables

**Table 1:** Group difference in creativity performance in paired-player mode, creative motivation, and self-efficacy.

	High-Performing Group		Low-Performing Group		*t*	*d*
	*Mean*	*SD*	*Mean*	*SD*		
CRAT group						
CRRAT	0.50	0.18	0.40	0.20	4.24 **	0.53
Intrinsic motivation	3.05	0.49	2.97	0.50	1.32	0.16
Extrinsic motivation	2.83	0.46	2.81	0.42	0.31	0.04
Self-efficacy	2.84	0.49	2.80	0.43	0.76	0.09
Divergent thinking group						
Fluency	16.54	6.57	13.60	5.96	3.80 **	0.47
Flexibility	8.53	2.74	7.45	2.25	3.50 **	0.43
Originality	15.12	8.58	11.65	7.57	3.47 **	0.43
Intrinsic motivation	3.05	0.49	2.96	0.49	1.55	0.19
Extrinsic motivation	2.81	0.47	2.84	0.40	−0.54	0.07
Self-efficacy	2.83	0.45	2.81	0.48	0.39	0.05

*Note.* ** *p* < 0.01. These results display performance in the paired-player mode.

**Table 2:** Correlations between creativity performance in paired-player mode, creative motivation, and self-efficacy.

	1	2	3	4	5	6
1. CRRAT	-					
Divergent thinking						
2. Fluency	0.18 **	-				
3. Flexibility	0.19 **	0.84 **	-			
4. Originality	0.08	0.88 **	0.72 **	-		
5. Intrinsic motivation	0.02	0.23 **	0.22 **	0.23 **	-	
6. Extrinsic motivation	0.03	0.07	0.07	0.04	0.11	-
7. Self-efficacy	0.03	0.19 **	0.13 **	0.18 **	0.68 **	0.10

*Note.* ** *p* < 0.01. These results display performance in the paired-player mode.

**Table 3:** Moderating effects of creative motivation and self-efficacy on divergent thinking and CRRAT.

	Divergent Thinking						CRRAT	
	Fluency		Flexibility		Originality			
	β	*t*	β	*t*	β	*t*	β	*t*
Group	0.42	3.56 **	0.38	3.24 **	0.38	3.23 **	0.51	4.21 **
CIM	0.07	0.85	0.09	1.10	0.07	0.80	0.00	0.04
Group × CIM	0.28	2.38 *	0.22	1.85 ^+^	0.28	2.38 *	−0.01	−0.05
*R*	0.34		0.31		0.33		0.25	
*R* ^2^	0.12		0.10		0.11		0.06	
*F*	11.30		9.31		10.32		5.94	
Group	0.46	3.84 **	0.43	3.54 **	0.42	3.49 **	0.51	4.21 **
CEM	0.07	0.77	0.03	0.28	0.02	0.17	0.04	0.42
Group × CEM	0.00	0.03	0.08	0.67	0.06	0.45	−0.03	−0.21
*R*	0.24		0.23		0.22		0.26	
*R* ^2^	0.06		0.05		0.05		0.07	
*F*	5.31		4.72		4.28		6.01	
Group	0.45	3.81 **	0.42	3.48 **	0.41	3.47 **	0.51	4.20 **
CSE	0.07	0.82	0.05	0.61	0.06	0.71	0.00	0.01
Group × CSE	0.25	2.07 *	0.16	1.31	0.25	2.09 *	0.04	0.29
*R*	0.32		0.26		0.30		0.26	
*R* ^2^	0.10		0.07		0.09		0.07	
*F*	9.67		6.17		8.57		6.01	

*Note.* ^+^ *p* < 0.10, * *p* < 0.05, ** *p* < 0.01.

## Data Availability

Data can be accessed by contacting the corresponding author.
